# A moodle course to substitute resuscitation teaching in a medical curriculum during the COVID-19 pandemic: A prospective pilot study

**DOI:** 10.3389/fpubh.2022.991408

**Published:** 2022-11-11

**Authors:** Florian Ettl, Christoph Schriefl, Jürgen Grafeneder, Dominik Gabriel Thallner, Matthias Mueller, Eva Fischer, Raphael Schlegel, Thorsten Sigmund, Michael Holzer, Sebastian Schnaubelt

**Affiliations:** ^1^Department of Emergency Medicine, Medical University of Vienna, Vienna, Austria; ^2^Department of Anaesthesiology, Clinic Donaustadt, Vienna Healthcare Group, Vienna, Austria; ^3^Department of IT-Systems and Communications, Medical University of Vienna, Vienna, Austria

**Keywords:** online education, online learning, resuscitation, teaching, medical education, distance learning, pandemic, COVID-19

## Abstract

**Background:**

Face-to-face medical education was restricted during the COVID-19 pandemic, leading to alternative teaching methods. Moodle^®^ (Modular Object-Oriented Dynamic Learning Environment) – an online course format – has not yet been sufficiently evaluated for its feasibility and effectiveness in teaching cardiopulmonary resuscitation.

**Methods:**

Medical students in the eighth semester took part in a Moodle^®^ course teaching basic life support, the ABCDE-approach, airway management, and advanced life support. The content was presented using digital background information and interactive videos. A multiple-choice test was conducted at the beginning and at the end of the course. Subjective ratings were included as well.

**Results:**

Out of 594 students, who were enrolled in the online course, 531 could be included in this study. The median percentage of correctly answered multiple-choice test questions increased after completing the course [78.9%, interquartile range (*IQR*) 69.3–86.8 vs. 97.4%, *IQR* 92.1–100, *p* < 0.001]. There was no gender difference in the median percentage of correctly answered questions before (female: 79.8%, *IQR* 70.2–86.8, male: 78.1%, *IQR* 68.4–86.8, *p* = 0.412) or after (female: 97.4%, *IQR* 92.1–100, male: 96.5%, *IQR* 92.6–100, *p* = 0.233) the course. On a 5-point Likert scale, 78.7% of students self-reported ≥4 when asked for a subjective increase in knowledge. Noteworthy, on a 10-point Likert scale, male students self-reported their higher confidence in performing CPR [female 6 (5–7), male 7 (6–8), *p* < 0.001].

**Conclusion:**

The Moodle^®^ course led to a significant increase in theoretical knowledge. It proved to be a feasible substitute for face-to-face courses – both objectively and subjectively.

## Introduction

During the first wave of the COVID-19 pandemic in the Spring 2020, all face-to-face teaching at the Medical University of Vienna (MUV) was suspended, including training in cardiopulmonary resuscitation (CPR) conducted by the Department of Emergency Medicine. Usually, the MUV human medicine curriculum features a variety of CPR education modules, ranging from basic life support (BLS) to complex advanced life support (ALS) scenario training (1, 2). However, the instructors were now faced with the challenge of ensuring consistency of at least BLS teaching despite continuous and repeated national lockdowns and strict meeting regulations. Information technology (IT) solutions for conferencing and learning have seen a boost in popularity since the pandemic and still partly substitute face-to-face teaching (3–8). Even though distance learning does also have its place in CPR training, under ideal conditions it cannot stand alone and must be accompanied by hands-on training (9). However, pre-course e-learning as part of a blended-learning approach is generally recommended (10, 11). In addition, the International Liaison Committee on Resuscitation advises video-only education when instructor-led training is not accessible (12).

The MUV uses the open-source software Moodle^®^ (Modular Object-Oriented Dynamic Learning Environment) to offer students time- and place-flexible e-learning throughout the curriculum (13, 14). Moodle^®^ has been used for CPR education before – mostly only in blended-learning environments – and has shown sufficient effectiveness and participant satisfaction (15–17).

To ensure that at least the essential resuscitation skills would be taught, a Moodle^®^ course with BLS and ALS content adapted from current guidelines (10, 18, 19) together with learning assessments, was developed and conducted. This pilot study aimed to assess the feasibility and effectiveness of a Moodle course in CPR education to provide evidence for future course adaptations.

## Materials and methods

This study was approved by the independent Ethics Committee of the Medical University of Vienna and complies with the Declaration of Helsinki. Given the COVID-19 restrictions at the time, establishing a control group was not possible as in-person teaching was prohibited by law. All participants provided written informed consent before study inclusion.

### Study population

As part of their mandatory curriculum, all medical students between the age of 18 and 65 in their eighth (out of 12) semester were included. Those students who did not finish the online course were excluded.

### Online course

A full description of the online course, including the used multiple-choice questions, can be found in the Supplementary material. In brief, the course consisted of four topics: (1) BLS, (2) ABCDE-approach, (3) airway management, and (4) ALS. In-depth learning material (e.g., videos and interactive quizzes) was provided.

### Data acquisition

The students had to complete an online course as part of their mandatory practical line element, “Reanimationsübungen II” (resuscitation training II). The course was conducted using the open-source software Moodle^®^ (Version 3.8.1).

Before the course started, a self-reported knowledge assessment concerning the course topics was performed. The rating on a 5-point Likert scale went from none to excellent knowledge. Furthermore, students were asked to rate their confidence in performing sufficient resuscitation on a 10-point Likert scale (very unconfident to very confident). Following the self-reported knowledge, a multiple-choice (MC_pre) test was performed. After completing the online course, the multiple-choice test was repeated (MC_post). Additionally, students were asked whether they thought that the online course led to a subjective increase in knowledge. The 5-point Likert scale ranged from “do not agree at all” to “fully agree.” The prospective data acquisition took place between March and May 2020.

### Statistical analysis

The results of MC_pre and MC_post were quantified as the percentage of correctly answered the questions.

Age was categorized due to the local data protection authority regulations to allow for anonymity. Categorical variables are summarized as counts and percentages and are compared using the χ^2^- or Fisher's exact test as appropriate. Continuous variables are expressed as mean and standard deviation (*SD*) or median and interquartile range (*IQR*) as applicable. Univariate differences between groups were assessed using the Mann–Whitney *U* test or Student's *t*-test as appropriate. In the case of parametric tests, normal distribution was assessed using the Kolmogorov–Smirnov test. To analyze the influence of gender and previous knowledge, a subgroup analysis was performed using an ANOVA.

No imputation for missing data was performed. Two-sided *p*-values of <0.05 indicated statistical significance. SPSS 23.0 (IBM Corporation, Armonk, NY, USA), R (Version 4.0.0), and GraphPad Prism 8.4.2 were used for all analyses.

## Results

A total of 594 students were enrolled in the online course. Of those, 575 (96.8%) gave informed consent. Additionally, 44 students did not complete the course, resulting in a sample size of 531 students.

Table 1 gives an overview of participants' age, gender, and previous knowledge. Given the small number of students specifying their gender as diverse (*n* = 4, 0.8%), gender aspects were only calculated for female and male students. Only 110 (20.7%) students did not specify any previous knowledge. The majority of students underwent their last resuscitation training in the previous year.

**Table 1:** Participants' age, gender, previous knowledge, and timepoint of last resuscitation training.

	**Total *n =* 531**
**Gender**, ***n*** **(%)**	
Female	299 (56.3)
Male	228 (42.9)
Diverse	4 (0.8)
**Age**, ***n*** **(%)**	
19–20	3 (0.6)
21–29	501 (94.4)
30–39	24 (4.5)
40–49	1 (0.2)
50–59	1 (0.2)
Not available	1 (0.2)
**Prior knowledge or training**, ***n*** **(%)**	
Nurse	10 (1.9)
Paramedic	148 (27.9)
No extracurricular training	373 (70.2)
**Last Resuscitation training**, ***n*** **(%)**	
The same year	33 (6.2)
The year before	381 (71.8)
2 years ago	77 (14.5)
≥2 years ago	37 (7.0)
Not specified	3 (0.6)

The self-reported knowledge about BLS assessed before the online course was reported favorably by the majority of the students [“good”: *n* = 241 (45.4 %); “excellent”: *n* = 92 (17.3%)]. For ALS, the results differed, with the majority stating to have “some” [*n* = 203 (38.2%)] or “intermediate” [*n* = 198 (37.3%)] knowledge. Similar results were found for the ABCDE-approach: “Some” knowledge was reported by 203 (38.2%), and “intermediate” by 198 (37.3%) students. The self-reported confidence in performing sufficient resuscitation was rated ≥7 by 284 (53.5%) students on a 10-point Likert scale.

**Figure 1:**
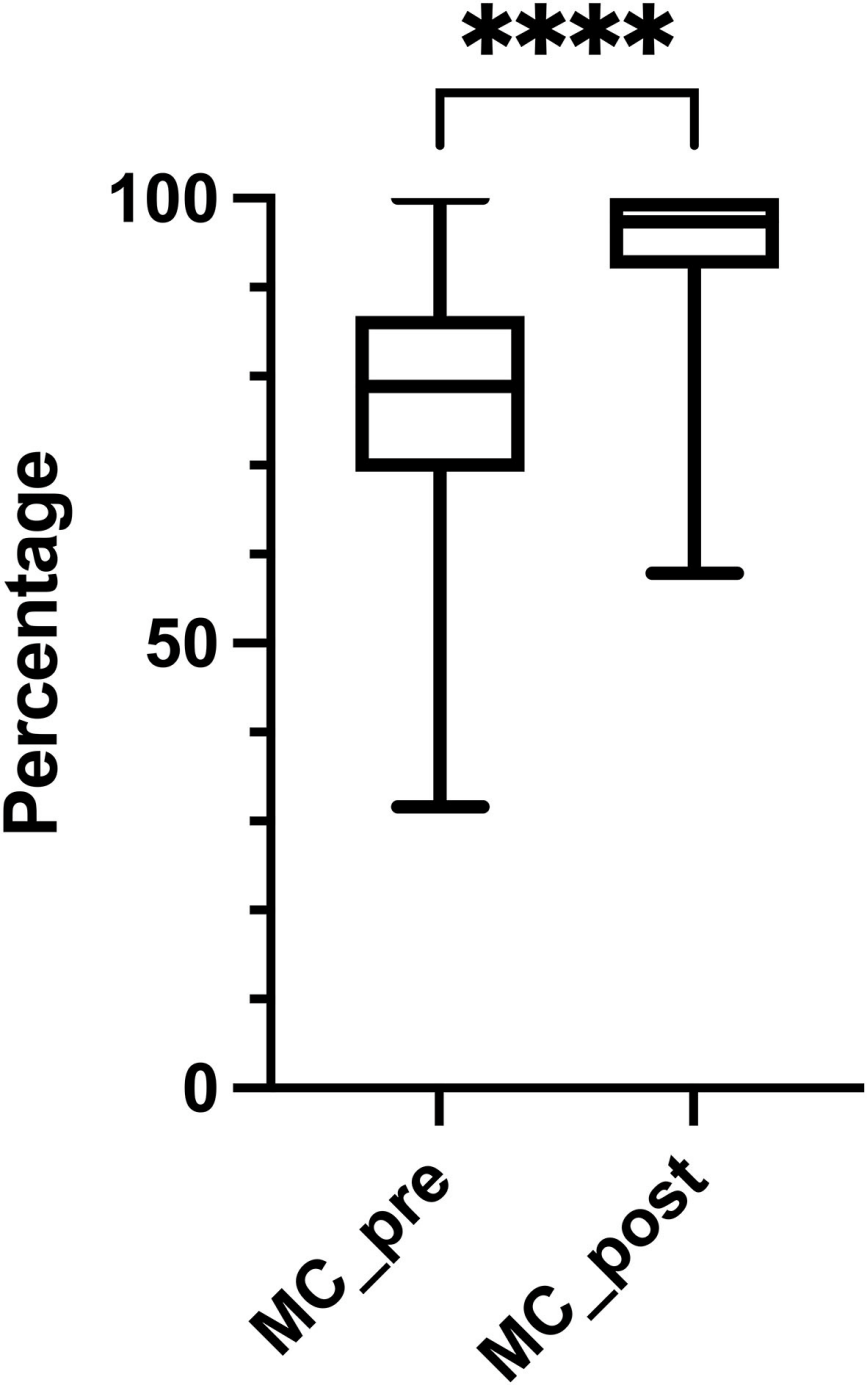
Knowledge gain. The median percentage of correctly answered multiple-choice test questions significantly increased after completing the course (78.9%, *IQR* 69.3–86.8% vs. 97.4%, *IQR* 92.1–100%, *p* < 0.001). MC_pre, multiple-choice test performed before the course; MC_post, multiple-choice test performed after the course. ****p < 0.001

Table 2 shows MC_pre and MC_post results. The median percentage of correctly answered multiple-choice test questions was significantly higher in the MC_post compared to the MC_pre (97.4%, *IQR* 91.2–100 vs. 78.9%, *IQR* 69.3–86.8, *p* < 0.001) (Figure 1). The rate of students with ≥90% correct answers increased from 17.3% before to 84.6% after the Moodle^®^ course. Students spent less time in completing the MC_post (6 min, *IQR* 4–11 vs. 8 min *IQR* 6–14, *p* < 0.001). On the 5-point Likert scale, 418 (78.7%) students reported ≥4 when asked for a subjective increase in knowledge.

**Table 2:** Median percentage of correctly answered multiple-choice test questions of the multiple-choice test carried out before (MC_pre) and after (MC_post) the Moodle^®^ (Modular Object-Oriented Dynamic Learning Environment) course.

	**MC_pre**	**MC_post**	
**Achieved MC test results**			
Median, % (IQR)	78.9 (69.3–86.8)	97.4 (92.1–100)	*p* < 0.001
Minimum, %	31.6	57.9	
Maximum, %	100	100	
**Time to complete test**, min (IQR)	8 (6–14)	6 (4–11)	*p* < 0.001

IQR, interquartile range; MC_pre, Multiple choice test conducted before the Moodle^®^ course; MC_post, Multiple choice test conducted after the Moodle^®^ course.

**Figure 2:**
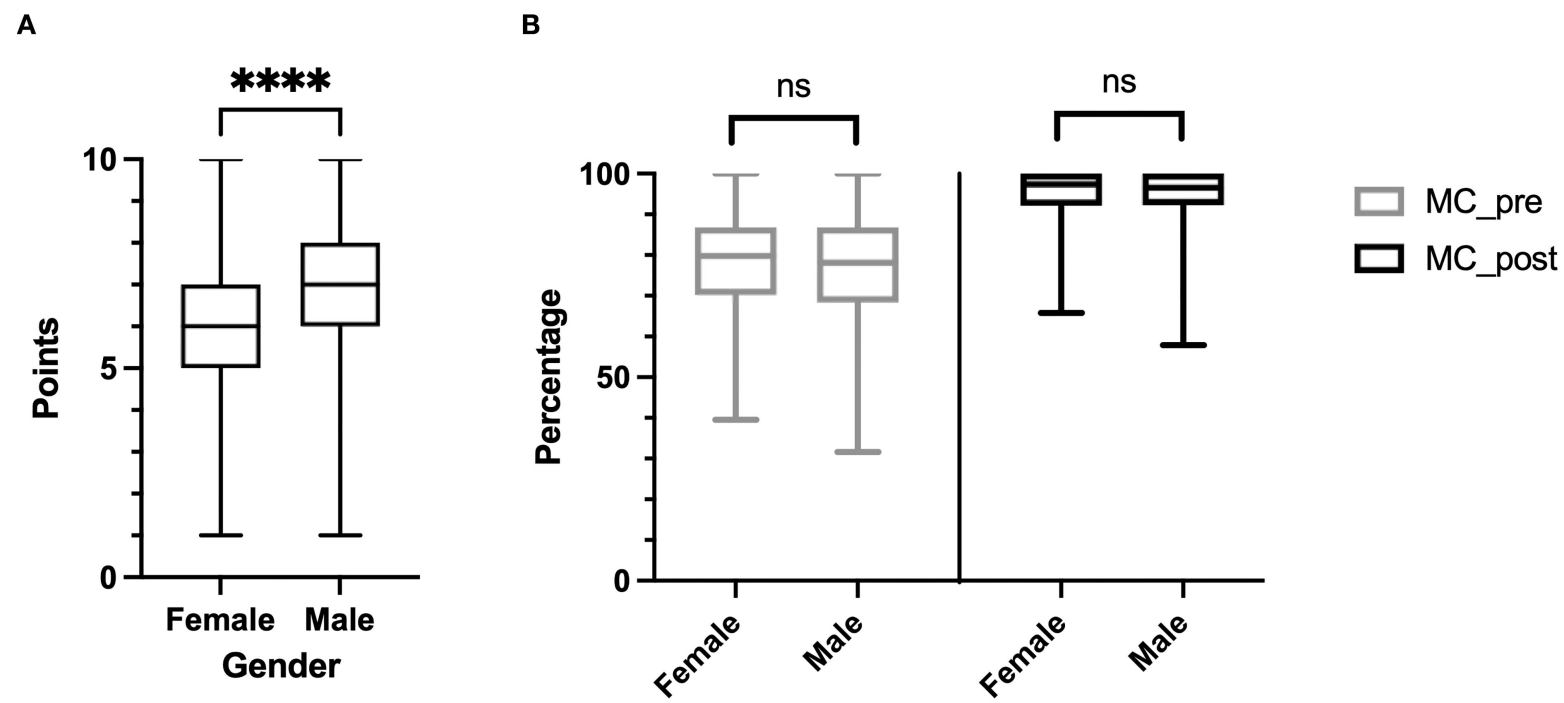
Gender differences. Male students self-rated their confidence in performing a sufficient resuscitation significantly higher **(A)**. There were no gender differences in the median percentage of correctly answered multiple-choice test questions performed before (MC_pre) and after (MC_post) the course **(B)**. ****p < 0.001; ns, not significant

Gender differences are presented in Table 3 and Figure 2. Although on a 10-point Likert scale, male students reported a higher confidence in performing a sufficient resuscitation (7 ± 1.9 vs. 5.99 ± 2.0 *p* < 0.001), the MC_pre test results did not differ significantly from female students (female: 79.8%, *IQR* 70.2–86.8 vs. male: 78.1%, *IQR* 68.4–86.8, *p* = 0.412). This was also true for the MC_post test (female: 97.4%, *IQR* 92.1–100 vs. male: 96.5% *IQR* 92.6–100, *p* = 0.233).

**Table 3:** Gender differences in the self-reported confidence in performing a sufficient resuscitation, as well as in MC_pre and MC_post results.

	**Female (*n =* 299)**	**Male (*n =* 228)**	
CPR confidence, points (IQR)	6 (5–7)	7 (6–8)	*p* < 0.001
MC-Test, % (IQR)			
MC_pre	79.8 (70.2–86.8)	78.1 (68.4–86.8)	*p* = 0.412
MC_post	97.4 (92.1–100)	96.5 (92.6–100)	*p* = 0.233

The confidence in performing sufficient resuscitation was assessed on a 10-point Likert scale. The result of the multiple-choice test was the percentage of correctly answered questions. CPR. cardiopulmonary resuscitation; MC, multiple choice; IQR, interquartile range; MC_pre, Multiple choice test conducted before the online course; MC_post, Multiple choice test conducted after the online course.

The results were similar for the confidence in performing a CPR (female: 5.99 ± 2.0, male: 7 ± 1.9, divers: 5 ± 0.8, *p* < 0.001). Furthermore, there was no significant difference in knowledge before (female: 79.8%, *IQR* 70.2–86.8; male: 78.1%, *IQR* 68.4–86.8; divers: 81.1%, *IQR* 70.2–90.8, *p* = 0.675) or after (female: 97.4%, *IQR* 92.1–100; male: 96.5%, *IQR* 92.6–100; Diverse: 93.9%, *IQR* 93–97.4, *p* = 0.675) the course.

Due to the large percentage of paramedics in the cohort (*n* = 148) and the large overlap of our course with the content of paramedic training, a sensitivity analysis was performed. Compared with no extracurricular training (*n* = 373), the median percentage of correctly answered multiple-choice test questions was significantly higher in the MC_pre test (82.9%, *IQR* 75.9–91.2 vs. 77.2%, *IQR* 66.7–85.1, *p* < 0.001), but not in the MC_post test (97.4%, *IQR* 94.7–100 vs. 96.5%, *IQR* 92.1–100, *p* = 0.11).

The time of the last resuscitation training showed a significant influence on the MC_pre test (*p* = 0.025, Table 4). The highest median was found in the group with the last resuscitation training within the last 12 months (86.8, *IQR* 76.3–94.7). However, there was no significant difference in the MC_post results (*p* = 0.131).

**Table 4:** Last resuscitation training and median percentage of correctly answered multiple-choice test questions of the multiple-choice carried out before the course (MC_pre) and after the course (MC_post).

**Last Resuscitation training**	***n*** **(%)**	**MC_pre median (IQR)**	**MC_post median (IQR)**
The same year	33 (6.2)	86.8 (76.3–94.7)	98.2 (94.7–100)
The year before	381 (71.8)	78.9 (69.3–86.8)	97.4 (92.1–100)
Two years ago	77 (14.5)	77.2 (68.4–84.2)	95.6 (91.2–100)
≥2 years ago	37 (7.0)	80.7 (66.7–88.6)	97.4 (94.7–100)
Not specified	3 (0.6)	80.7 (73.7–84.2)	100 (100–100)

## Discussion

In this study, we showed that using an online Moodle^®^ course as a substitute for an in-person resuscitation teaching resulted in an objective and subjective knowledge gain. Noteworthy, interactive learning videos were cited as helpful for the learning progress.

### The need for substitutional educational elements

Perkins et al. (20, 21) were able to show that supplementary and substitute e-learning leads to the same results of a knowledge test as conventional teaching methods (20, 21). An improvement in performance has not yet been shown, still leaving potential room for various improvements of respective elements of digital teaching. Ultimately, the educational goal of resuscitation teaching must be the improvement of practical skills leading to better patient outcomes such as survival or favorable neurological outcome (22). However, out of simple necessity and lack of other possibilities, the ongoing global pandemic took digital teaching to the next level. Especially in medical education, e-learning is now increasingly used in all areas either as a substitute for impossible face-to-face teaching or as an addition to traditional course formats (23, 24). In our study, we were able to show that in an exceptional situation such as a pandemic, an online course is at least suitable as a theoretical alternative program for resuscitation training. Naturally, known advantages of e-learning also apply: it is flexible in both time and location for participants, cost efficiency, a low number of instructors needed, and better standardization capability have been reported in prior studies (25).

### Moodle^®^ as an excellent platform for knowledge transfer

The previous literature shows that in pre- and post-test evaluations, the performance after online courses is at least as good as after “offline” education – sometimes even better (21). Accordingly, we could show that in the final test, the number of students who achieved 90% or more concerning correct answers increased to an impressive 84.6% (*n* = 449) as compared to 17% (*n* = 92) in the entry test. Of note, 458 participants (86.3%) were able to improve their results by more than 5%.

Also, the evaluation of the subjective assessment of the course showed highly positive feedback: A large number of students (78.7%) were able to gain a subjective increase in knowledge. Especially the interactive videos were appreciated and classified as helpful. These results are consistent with the previous data (20) and support the assumption that students approve this form of teaching.

### Prior CPR knowledge – a debatable influencer on educational outcomes?

No significant influence of prior knowledge on learning success could be found in the present study. It is possible that the effect of prior knowledge could not be sufficiently differentiated in the MC_post test due to the generally very high number of correct answers in all groups. In contrast, Thorne et al. have shown a better performance of study participants with previous ALS experience; however, the authors had included significantly more highly qualified healthcare personnel, doctors, and nurses with clinical experience in an ALS course (26). The participants in our present study are mid-career medical students who had received little previous ALS training. Naturally, not only the level of prior knowledge and education but also the frequency of practical application and the timeframe since the last course would be of importance to the susceptibility to and outcomes of a further course on the topic. As we have not assessed this, the optimal online course for each level and timeframe of CPR education remains a knowledge gap. The time since the last CPR training did not affect course performance. By the end of the course, all groups were brought to the same level of knowledge, and the learning objectives were adequately achieved.

### Gender differences

None of the so-far mentioned previous literature included a gender-related evaluation. The gender “diverse” was rarely selected, so only descriptive analyses were conducted in this regard. Male participants self-rated their own skills and knowledge better than women but did equally well in the multiple-choice test. This effect of the more positive self-assessment among men has already been reported (27). Of note, our results did not show any significant influence of gender on learning success, and there was no difference in the MC test results.

### Generalizability and future prospects

To our knowledge, this study is one of the largest to evaluate an online Moodle^®^ course and its learning success in this field. Due to the high number of participants and the low drop-out rate, we consider our study to generally represent students at the given level of education.

The described course follows the recommendations of the current European Resuscitation Council (ERC) guidelines to use e-learning as an alternative teaching method (10). Further studies should examine the effect of a Moodle^®^ course on additional outcomes such as skills. Moreover, it would be essential to evaluate which course content particularly motivates students or contributes to learning success. With respective feedback, courses like this can be further optimized and, with appropriate preparation, the full possibilities of digital teaching can be utilized. Of importance, to fully evaluate the educational potential of our approach, a randomized controlled trial with comparison groups of face-to-face and blended learning options must be conducted. Finally, considering the problem that skills and knowledge, which are achieved within conventional course models, tend to fall into oblivion as time passes by (22) a Moodle^®^ course could in the future be assessed for its value for refresher and retention approaches.

### Limitations

Due to the ongoing pandemic and the – at least intermittent – ban on face-to-face teaching, a control group (teaching in attendance) was impossible. Testing of practical skills also had to be omitted for this reason. Also, due to the acute necessity to provide at least any resuscitation education, it was necessary to react quickly, and the online course had to be created in a very short period of time. For this reason, resources were limited and the full possibilities of digital teaching could not be exploited.

The multiple-choice test before and after the course was identical. They contained the same questions to avoid a possible bias in terms of difficulty. This was, of course, a limitation, as the questions were already known at the second assessment. However, no feedback had been given on the answers submitted during the initial knowledge check.

This study could not assess the impact of the Moodle course on practical skills and performance in real life. This limitation – due to the ongoing pandemic at the time of the study – needs to be addressed by future studies. Nevertheless, acquiring theoretical background *via* the Moodle^®^ course before seminars might allow more time for practical training and thus also have a potential impact on real-life performance.

An online examination that students carry out alone relies on students' honor. This course format bears the well-known risk that students use aids or help each other. However, any dishonesty was likely the same for the test before and after the online course. Thus, knowledge gain most likely caused the increase in the students' median test results. Nevertheless, to prevent cheating and to appeal to students' honor, we informed the students that the number of correct answers did not affect their grades and that the only purposes were self-examination and scientific evaluation.

## Conclusion

An online Moodle^®^ course led to a significant increase in theoretical knowledge on cardiopulmonary resuscitation among medical students. Although face-to-face teaching elements in resuscitation education should be performed whenever possible, the evaluation of new digital- or blended learning elements and -approaches seems crucial in times of global pandemics and a call for cost-efficiency. Also, a Moodle^®^ course seems feasible as a substitute for other course designs that are temporarily not practicable or available.

## Data availability statement

The raw data supporting the conclusions of this article will be made available by the authors, without undue reservation

## Ethics statement

The studies involving human participants were reviewed and approved by the Ethics Committee of the Medical University of Vienna. The patients/participants provided their written informed consent to participate in this study.

## Author contributions

FE, CS, DT, EF, RS, and SS contributed to data acquisition and study design. FE, JG, DT, and SS drafted the manuscript and executed data analyses. MM, EF, RS, TS, and MH contributed to study design and further amended the manuscript. MH supervised the study process. All authors critically revised and approved the final version of the manuscript.

## Conflict of interest

Author SS is ILCOR EIT Task Force Member. The remaining authors declare that the research was conducted in the absence of any commercial or financial relationships that could be construed as a potential conflict of interest.

## Publisher's note

All claims expressed in this article are solely those of the authors and do not necessarily represent those of their affiliated organizations, or those of the publisher, the editors and the reviewers. Any product that may be evaluated in this article, or claim that may be made by its manufacturer, is not guaranteed or endorsed by the publisher.
