# Supplementary material for: A moodle course to substitute resuscitation teaching in a medical curriculum during the COVID-19 pandemic: A prospective pilot study
Source: Front Public Health. 2022 Nov 11;10:991408. doi: 10.3389/fpubh.2022.991408 (PMC9691759; doi:10.3389/fpubh.2022.991408)
Supplement: Supplementary file 1 [file Data_Sheet_1.DOCX]

Supplementary Material

# Description of the used Moodle® course

Due to the pandemic, „Resuscitation Training II“ had to be taken as an online Moodle course. The course is divided into different topics: “Introduction”, “Basic Life Support (BLS) and Automated External Defibrillator ( AED) – Rehearsal”, the “ABCDE Mnemonic”, “Airway Management”, “Advanced Life Support (ALS)” and “Final Test”. The students have to work on these one by one as the contents are based on one another – knowledge is supposed to evolve throughout the course.

The section “Introduction” gives information about the modalities of the course including the final test. Furthermore, information about data protection is provided and students are about to sign an informed consent in which they can accept or refuse to participate in this study. It is emphasized that their decision will not have an impact on their assessment. In this section, the initial checkup is to be found as well as the entry questionnaire, which does not have an influence on the final assessment. In addition, students are asked to participate in a poll on the probability of successful CPR. The consecutive section involves content students are familiar with. BLS and AED guidelines should be revised. This section offers an interactive video on BLS and AED use. In another interactive video, students should assess the quality of given chest compressions. The section “ABCDE mnemonic” includes one interactive video which shows how this approach can be applied in real time. Two quizzes help the students to revise the theory of the ABCDE approach. In the section “Airway Management” students are virtually introduced to the different forms of airway devices and techniques to secure the airway. A variety of photographs that are accompanied by an explanation introduce the students to the bag mask ventilation. Interactive videos demonstrate the correct performance and usage of the EC Clamp, the Jaw Thrust Maneuver, oropharyngeal and nasopharyngeal tubes as well as the laryngeal tube. Finally, this section offers a quiz on the respective airway devices. The section on ALS provides the ALS guidelines including the algorithm. Two interactive tools make the students work on medication and reversible causes. Two videos give examples for shockable and non-shockable rhythms. In the section “Final Test” the students have to do the final exam which equals the initial checkup – the students have one try but there is no time limitation. The final survey enables the students to give feedback.

# Used test questions

**General entry questions**

1. What is your gender?
   1. diverse
   2. female
   3. male
2. Which age group are you in?
   1. 17-18 years
   2. 19-20 years
   3. 21-29 years
   4. 30-39 years
   5. 40-49 years
   6. 50-59 years
   7. 60 years or older
3. What is the highest level of education you successfully completed?
   1. Upper secondary school or vocational school with respective school leaving examination *(with examples of the respective German expressions for the schools)*
   2. University
   3. College of higher education
   4. Compulsory school and/or vocational school plus additional university entrance exam
4. Have you already completed a course / attended training in which resuscitation was part of the curriculum? (besides the respective elements that are part of the medical studies) Please choose the most valuable education.
   1. Emergency medical technician
   2. Paramedic
   3. „Rettungsassistent“ *(a special form of an advanced preclinical paramedic training in Germany)*
   4. Nurse
   5. Other forms of nursing education
   6. International course formats (e.g., ERC course)
   7. Elective Courses (e.g., through other organizations)
   8. Other
5. When was your last CPR training (if any)?
   1. This year
   2. Last year
   3. 2 years ago
   4. More than 2 years ago
6. How would you assess your own knowledge concerning Basic Life Support?
   1. I have none
   2. rather poor
   3. moderate
   4. good
   5. excellent
7. How would you assess your own knowledge concerning Advanced Life Support?
   1. I have none
   2. rather poor
   3. moderate
   4. good
   5. excellent
8. How would you assess your own knowledge concerning the ABCDE approach?
   1. I have none
   2. rather poor
   3. moderate
   4. good
   5. excellent
9. How would you assess your airway management skills?
   1. I have none
   2. rather poor
   3. moderate
   4. good
   5. excellent
10. At present, what is your level of confidence when providing CPR?
    1. 1 (highly insecure)
    2. 2
    3. 3
    4. 4
    5. 5
    6. 6
    7. 7
    8. 8
    9. 9
    10. 10 (highly confident)

*A poll on the probability of successful CPR:*

1. How high do you think is the approximate probability of surviving an Out-of-Hospital Cardiac Arrest (OHCA) in Vienna?
   1. approx. 2%
   2. approx. 5%
   3. approx. 12%
   4. approx. 45%
   5. approx. 50%

**Initial and final test**

1. How do you correctly check for breathing in an unresponsive person?
   1. I hold the person’s nose.
   2. To smell.
   3. For a maximum of 10 seconds (right)
   4. Listen (right)
   5. Look towards the chest (right)
2. What do you consider when giving chest compressions (adult patient)?
   1. I ensure full chest recoil. (right)
   2. I make sure that the patient is placed on hard ground. (right)
   3. I compress the chest using one hand.
   4. I position my shoulders directly over my hands. (right)
   5. I perform chest compressions with flexed elbows.
3. Which rate would you choose when providing chest compressions in an adult person?
   1. 140-180/min
   2. 80-100/min
   3. 120-140/min
   4. 60-80/min
   5. 100-120/min (right)
4. What is the correct depth an adult’s chest should be compressed in case of CPR?
   1. 3-4cm
   2. One tenth of the rib cage
   3. 5-6cm (right)
   4. 6-7cm
   5. 4-5cm
5. You use an Automated External Defibrillator (AED). After the rhythm analysis, a shock is advised. How is defibrillation performed correctly?
   1. After the shock is delivered, I immediately continue providing CPR. (right)
   2. In case an AED is used, the shock must not be necessarily delivered.
   3. After shock delivery, I immediately check on breathing and circulation.
   4. I make sure no one is touching the patient. (right)
   5. I shout out loudly: “Everyone, take a step back! Attention, shock will be delivered!“ (right)
6. What does the “A“ stand for in the ABCDE mnemonic?
   1. Alert
   2. Airway (right)
   3. Auscultation
   4. Address
   5. Assessment
7. What does the “D” stand for in the ABCDE mnemonic?
   1. Desensibility
   2. Deescalation
   3. Disability (right)
   4. Delirscreening
   5. Debriefing
8. What does the “E” stand for in the ABCDE mnemonic?
   1. Exit
   2. Expedition
   3. Expenditure
   4. Exposure (right)
   5. End-of-life
9. What procedures have to be performed when it comes to “B” when using the ABCDE mnemonic?
   1. Measure respiratory rate. (right)
   2. To do a stress ECG.
   3. Lung auscultation. (right)
   4. To perform a spirometry.
   5. To measure oxygen saturation (SpO2). (right)
10. What procedures have to be performed when it comes to “D” when using the ABCDE mnemonic?
    1. Assessment of patient’s orientation (right)
    2. Endoscopy of the ocular fundus
    3. EEG
    4. To check the blood sugar (right)
    5. Assessment of mental/neurological status (right)
11. Which of the following are to be labeled as a supraglottic airway device?
    1. Oropharyngeal Tube (right)
    2. Müller-Tube
    3. Nasopharyngeal Tube (right)
    4. Laryngeal Tube (right)
    5. Endotracheal Tube
12. Which useful maneuvers in order to open the airway do you know?
    1. ABCDE-Grip
    2. Babinski-Maneuver
    3. Jaw Thrust Maneuver (right)
    4. Head Tilt Chin Lift Maneuver (right)
    5. Heimlich Maneuver
13. A patient with an altered state of consciousness in supine position produces an inspiratory breath sound. The oxygen saturation is not within the acceptable range. What could you do next?
    1. I would immediately perform a surgical tracheostomy.
    2. I would immediately give mouth-to-mouth ventilation.
    3. I would place an oropharyngeal tube. (right)
    4. I would perform the Jaw Thrust Maneuver. (right)
    5. I would place a nasopharyngeal tube. (right)
14. You want to ventilate a patient, but you fail to maintain an airtight seal. Which maneuvers could help you?
    1. The use of a bigger bag.
    2. The two-handed, two-person technique. (right)
    3. Press the bag’s whole content into the patient’s lungs.
    4. To cut a hole into the mask.
    5. The placement of an oropharyngeal tube. (right)
15. Which Heart Rhythms are shockable?
    1. AV-Block
    2. Pulseless electrical activity (PEA)
    3. Ventricular fibrillation (VF) (right)
    4. Asystole
    5. Pulseless ventricular tachycardia (pVT) (right)
16. When should Adrenaline be administered during CPR?
    1. In case of a non-shockable rhythm as soon as possible. (right)
    2. After delivery of the third shock. (right)
    3. After delivery of the fifth shock. (right)
    4. 3-5 minutes after initiation of CPR.
    5. After the first shock.
17. How much Adrenaline should be administered during CPR?
    1. 1 mg (right)
    2. 10 mg
    3. 20 mg
    4. 0.5 mg
    5. 0.01 mg
18. At which point during CPR should Amiodarone be administered?
    1. After the delivery of the first shock.
    2. About 3-5 minutes after initiation of CPR.
    3. In case of non-shockable rhythms as soon as possible.
    4. After delivery of the third shock. (right)
    5. After endotracheal intubation is done.
19. When do you check the pulse during CPR?
    1. I need to check the pulse in order to recognize PEA or pVT correctly. (right)
    2. When I perceive a rhythm that could produce normal circulation, I will check the pulse. (right)
    3. At every second rhythm check I check the pulse.
    4. I never check the pulse.
    5. I always check the pulse during defibrillation.

**General final questions**

1. This course was a positive contribution to my knowledge acquisition.
   (1 = absolutely, 5 = not at all)
   1. 1
   2. 2
   3. 3
   4. 4
   5. 5
2. Due to this course I feel well prepared for future practical lessons and practical tasks. (1 = absolutely, 5 = not at all)
   1. 1
   2. 2
   3. 3
   4. 4
   5. 5
3. Because of this course, I feel able to perform CPR.
   (1 = absolutely, 5 = not at all)
   1. 1
   2. 2
   3. 3
   4. 4
   5. 5
4. I am using Moodle on the following devices:
   1. Laptop
   2. Tablet
   3. Smartphone
   4. PCs provided by the Medical University of Vienna
   5. Other
5. What is the estimated time you spent on this course (in hours)?
   1. 0-1
   2. 1-2
   3. 2-3
   4. 3-4
   5. 4-5
   6. 5-6
   7. 6-7
   8. >7
6. Is the Moodle Learning Platform suitable for your needs?
   1. Yes, it is supportive concerning my learning progress.
   2. Yes, I am using it as an addition to my self-study.
   3. No, I am just using it, because it is mandatory.
   4. No, for me, Moodle is a waste of time.
   5. I am not capable of evaluating its suitability.
7. The tutorial videos ...
   1. I watched them several times.
   2. I watched them and rehearsed specific scenes.
   3. I just glanced over them.
   4. I did not really consciously watch them.
   5. I perceived them as being useful.
   6. I did not perceive them as being useful.
8. The tutorial videos…
   1. contributed to the learning success.
   2. did not contribute to the learning success.
   3. are useful in order to prepare for possible hands-on classes.
   4. are not useful in order to prepare for possible hands-on classes.
9. Information about the course’s content was shared with other students as well.
   1. Yes
   2. No
10. This course in general was talked about or worked on together with other students as well.
    1. Yes
    2. No
11. A Moodle course cannot completely replace a CPR hands-on training. How many % of your personal learning objectives for “Reanimationsübungen II” did you achieve? (please insert % from 0 to 100)
    1. „Text“
